# Impact of transcutaneous vagus nerve stimulation on healthy cognitive and brain aging

**DOI:** 10.3389/fnins.2023.1184051

**Published:** 2023-07-28

**Authors:** Erin Trifilio, Destin Shortell, Sarah Olshan, Alexandria O’Neal, Jozee Coyne, Damon Lamb, Eric Porges, John Williamson

**Affiliations:** ^1^Center for OCD and Anxiety Related Disorders, Department of Psychiatry, McKnight Brain Institute, College of Medicine, University of Florida, Gainesville, FL, United States; ^2^Brain Rehabilitation and Research Center, Malcom Randall VAMC, Gainesville, FL, United States; ^3^Department of Clinical and Health Psychology, College of Public Health and Health Professions, University of Florida, Gainesville, FL, United States; ^4^Center for Cognitive Aging and Memory, McKnight Brain Institute, University of Florida, Gainesville, FL, United States

**Keywords:** tVNS, aging, vagus, transcutaneous vagus nerve stimulation, inflammation, brain, autonomic, cognitive

## Abstract

Evidence for clinically meaningful benefits of transcutaneous vagus nerve stimulation (VNS) has been rapidly accumulating over the past 15  years. This relatively novel non-invasive brain stimulation technique has been applied to a wide range of neuropsychiatric disorders including schizophrenia, obsessive compulsive disorder, panic disorder, post-traumatic stress disorder, bipolar disorder, and Alzheimer’s disease. More recently, non-invasive forms of VNS have allowed for investigations within healthy aging populations. These results offer insight into protocol considerations specific to older adults and how to translate those results into effective clinical trials and, ultimately, effective clinical care. In this review, we characterize the possible mechanisms by which non-invasive VNS may promote healthy aging (e.g., neurotransmitter effects, inflammation regulation, functional connectivity changes), special considerations for applying non-invasive VNS in an older adult population (e.g., vagus nerve changes with age), and how non-invasive VNS may be used in conjunction with existing behavioral interventions (e.g., cognitive behavioral therapy, cognitive training) to promote healthy emotional and cognitive aging.

## Introduction

To understand how transcutaneous vagus nerve stimulation (tVNS) may promote healthy cognitive aging, it is imperative to understand the landscape of the aging brain. Some cognitive domains are known to decline with age while others are held relatively constant. Processing speed and episodic memory typically decline with advanced age, even in the absence of mild cognitive impairment (MCI) or dementia. However, verbal skills such as vocabulary and overall intelligence (i.e., I.Q.) tend to remain constant throughout the lifetime (Salthouse, 2019). Most cognitive aging interventions focus on improving and/or maintaining performance in the typically declining domains of processing speed and memory. The history of intervention to improve cognitive aging is extensive and it not be reviewed in depth here. However, we do introduce conceptual terminology that is well-accepted in the field of cognitive neuroscience and is often the basis for cognitive intervention studies in older adults.

The primary goals of healthy cognitive aging interventions are well-articulated by Cabeza et al. (2018) as the concepts of cognitive “maintenance” and “compensation.” “Maintenance” is defined as “the preservation of neural resources, which entails ongoing repair of the brain” and “compensation” as “the cognition-enhancing recruitment of neural resources in response to relatively high cognitive demand.” We define maintenance in the context of tVNS as the effectiveness of tVNS in upregulating or initiating neural repair and plasticity mechanisms. As discussed in Cabeza et al., this can occur on several levels including different aspects of the brain (e.g., gray matter or white matter), neurotransmitter functions, or different brain regions (e.g., hippocampus, prefrontal cortex). This definition of maintenance is most easily quantified using longitudinal study designs. Compensation in the context of tVNS is best defined by the effectiveness of tVNS to either upregulate existing, select different, or reorganize to engage different neural mechanisms to improve performance on cognitive tasks. In this way, compensation in response to tVNS may be most easily studied using functional neuroimaging techniques such as functional magnetic resonance imaging (fMRI) in conjunction with cognitive testing over shorter time frames.

These concepts well describe the cognitive benefits that tVNS may provide to the aging brain and provide a useful structure for discussing the possible mechanisms and biomarkers of the effectiveness of tVNS in promoting healthy cognitive aging. While tVNS has been studied as an intervention for other aspects of healthy aging including quality of life, autonomic function, and mood, this review will focus primarily on promotion of healthy cognitive/brain aging. This will be discussed by reviewing the effects of tVNS in both healthy older adults and animal models of healthy aging and how these mechanisms may facilitate healthy cognitive aging as defined by maintenance or compensation. We will also discuss specific considerations for vagus nerve stimulation in an aging population including physiological changes to the vagus nerve with age.

## Vagus nerve and implications for cognitive aging

The vagus nerve is the tenth cranial nerve, named for the Latin word meaning “wandering” due to its extensive afferent and efferent projections throughout the body. It is comprised of approximately 20% efferent and 80% afferent fibers. The efferent or motor visceral pathways innervate almost all organs below the neck, including the heart, lungs, and gastrointestinal tract. The afferent pathway projects to cortical and subcortical structures in the brain, including the hippocampus, thalamus/hypothalamus, insula, prefrontal cortex, and motor cortex. fMRI studies have shown that stimulation of the vagus nerve induces activity in the locus coeruleus (LC) and other cortical and subcortical regions including the caudate, anterior cingulate, thalamus, hypothalamus, and frontal cortices (Frangos et al., 2015; Yakunina et al., 2017; Badran et al., 2018; Yakunina et al., 2018). Importantly, there are four brainstem nuclei associated with the vagus nerve: the nucleus tractus solitarii (NTS), nucleus ambiguus (NA), dorsal motor nucleus of the vagus (DMN), and the spinal trigeminal nucleus (STG).

The NTS afferent pathway is of special interest as tVNS is hypothesized to influence a broad spectrum of neurotransmitters and neuromodulators important for cognition via the NTS, including noradrenaline/norepinephrine (NA), dopamine, serotonin, gamma-aminobutyric acid (GABA), and acetylcholine (Huffman et al., 2019; Broncel et al., 2020). The NTS also has influence over the function of brain structures that are implicated in declining cognition and neurodegenerative disorders, such as the LC and hippocampus. The LC is of particular importance in common causes of unhealthy aging such as in neurodegenerative disorders. In Alzheimer’s disease progression, the LC is one of the earliest sites of AD pathology (Mather and Harley, 2016; Matchett et al., 2021). It is important to note that AD pathology is present at increasing levels with age and in older ages is statistically normal in prevalence; it is also the case that AD pathology may be present without clinical manifestation, meaning in cognitively healthy older adults. The LC is the brain’s primary source of NA and is an important component of the ascending noradrenergic pathway. This system is critical for many functions affected by neurodegenerative disease and brain aging, including memory formation (Mello-Carpes and Izquierdo, 2013; Sara, 2015; Suarez et al., 2018; James et al., 2020), attention and working memory (Unsworth and Robison, 2017), arousal (Berridge, 2008), sleep (Aston-Jones et al., 2001), stress (Wood and Valentino, 2017), neuroendocrine functions (Myers et al., 2017), and other cognitive functions (Weitekamp and Hofmann, 2014). Specifically in neurodegenerative disease, the LC-noradrenergic system modulates several processes that are altered in the brains of patients at risk for AD, including synaptic plasticity, inflammation, metabolism, and blood-brain-barrier permeability (Mravec et al., 2014). The LC also projects to the CA1 fields of the hippocampus, an area heavily impacted by AD pathology and critical for memory formation (De Flores et al., 2015). Several studies of patients with AD have found decreased activation in the medial temporal lobe during episodic encoding tasks (Dickerson and Sperling, 2008), and hippocampal atrophy is an early marker of AD pathology (Van de Pol et al., 2006). The function of the hippocampus may be modifiable with tVNS via the auricular branch of the vagus nerve and relevant projections to the LC and hippocampus from the NTS.

## Vagus nerve and aging

An important consideration for tVNS research in aging populations is how vagus nerve structure and function change with age. Ultimately, limited research exists on age-related changes in vagus nerve structure and function, making conclusions unclear.

Previous research in rats has demonstrated age-related changes in the structure of vagal visceral terminals synapsing on muscle wall and gastrointestinal tract mucosa. These changes were associated with age-related structural changes in the target organs, suggesting that the trophic environment of afferents may affect their structural integrity (Phillips et al., 2010). These results highlight the importance of considering the health of associated organs when studying branches of the vagus nerve in healthy older adults.

Several other studies in rodents have shown structural differences in the vagus nerve and associated structures that may suggest differential functionality with increased age. A study of mice across a spectrum of ages (i.e., 6, 15, 25, 28, and 31 months) compared the preganglionic vagus fibers in the dorsal motor nucleus and nucleus ambiguus. The number of neurons in the dorsal motor nucleus was significantly lower in mice 25 months and older, while sections of the nucleus ambiguus had a significantly lower neuron count in mice 15 months and older. Neuronal nuclear diameter in the nucleus ambiguus did not change with age, while the neuronal nuclear diameter of the dorsal motor nucleus of the vagus increased after 25 months of age (Sturrock, 1990). Another study compared the total number and density of axons projecting from the nucleus ambiguus to the atria and the density of synaptic-like endings between young and aged rats, all of which were reduced in aged rats. Moreover, structural changes occurred in many of the axons and terminals of aged rats, including swollen or bulging segments, fewer terminals, and swollen terminal varicosities. These changes may reflect degeneration or retraction of axons and terminals with age (Ai et al., 2007).

While these animal model studies suggest some age-related structural changes may occur in the vagus nerve, it is unclear if functional changes occur with age in animal models or if structural changes impact vagus nerve function with age. One study investigated how conduction velocities of myelinated and unmyelinated vagus nerve fibers changed with age in rats aged 28–920 days. In myelinated fibers, conduction velocities increased up to 200–300 days old after which they remained largely stable until they slightly decreased around 700–900 days old. Unmyelinated fiber conduction velocities increased up to 200 days old after which they remained largely stable with age (Sato et al., 1985).

Investigation of age-related structural and functional changes in human vagus nerves are similarly limited. A cross-sectional study in 279 human cadavers examined the total number of nerve fibers and the average area of nerve fibers in 30 specimens of the vagus nerve. While the total number of vagus nerve fibers in cadavers was not significantly associated with age, the transverse area of myelinated vagus nerve axons decreased with increasing age (*r* = −0.74). Cadaver ages ranged from the mid-30s to late 90s; however, most of the cadavers were older adults (65+). Thus, results should be interpreted with caution (Moriyama et al., 2007). Another cadaver study investigated the morphometry and vascularity of the cervical vagus nerves of 27 individuals (mean age at death = 88.1 ± 6.6 years, range = 73–103 years). Most notably, they found age was negatively associated with average cross-sectional area with a small effect (*r* = −0.28) but not with other measures of nerve morphometry (Hammer et al., 2018).

*In vivo* investigations of age-related changes in the human vagus nerve are also underway. In one study, 70 adult individuals (age range approximately 20s to 80s) underwent high-resolution ultrasound to examine age-related changes in the cross-sectional area of the bilateral vagus nerves at the mid-cervical level. The cross-sectional areas of the right and left vagus nerves were not significantly associated with age (Walter and Tsiberidou, 2019). Another study using high-resolution ultrasound in 60 healthy individuals (mean age = 49.7 ± 19.7 years, range = 22–76 years) found age was significantly negatively associated with right proximal (*β* = −0.35) and left distal (*β* = −0.39) vagus nerve cross-sectional areas with a small to moderate effect size. The multilinear regression model also controlled for gender, body weight, and height. Right distal (*β* = −0.27) and left proximal (*β* = −0.18) cross-sectional areas were not significant in the model (Pelz et al., 2018).

Another study recruited 20 younger (26–30 years) and 20 older adults (51–55 years) to examine differences in vagal structure and function between the two groups. Right, left, and average vagus nerve cross-sectional areas were not significantly different between the two age groups. The older group demonstrated slower vagus somatosensory evoked potential (VSEP) latencies (Laucius et al., 2021). These VSEP results partially replicated previous research, which also demonstrated slower VSEP latencies in older adults (51– 73 years) versus younger participants (20–40 years) (Fallgatter et al., 2005). Many papers and reviews have characterized age-related changes in vagus nerve function through heart rate variability (HRV) measurements (Jandackova et al., 2016; Geovanini et al., 2020; Thayer et al., 2021). These studies seem to suggest HRV decreases with age after postpubertal development and may increase in older adulthood. Overall, evidence for age-related changes in human vagus nerve structure is mixed. Additional research outside of heart-rate variability is needed to determine if age-related functional changes occur in different systems impacted by the vagus nerve.

Because the vagus nerve may exhibit age-related structural and functional changes, verification of vagal engagement during tVNS is crucial to ensure the validity of the stimulation and the accuracy of results. Even in the absence of age-related changes to the vagus nerve, age-related changes in the cardiovascular system are well documented (Wei, 1992). While speculative, these changes could impact vagal projections through compression or other means. Similarly, aging-related diseases may also cause changes in vascular systems or target organs. Notably, vascular diseases (e.g., diabetes, hypertension, hyperlipidemia) and peripheral neuropathies are common with age such that they are often not excluded for in studies of healthy cognitive aging. Additional research is necessary to understand what role aging and age-related diseases may play in vagus nerve structure and function.

## Neurotransmitter impacts

The vast majority of studies investigating the effects of tVNS on cognition are from the past 5 years. Most have been conducted in young and healthy individuals. Though findings across studies implicate a vast array of cognitive domains, many can be conceptualized in terms of the neuroanatomical underpinnings of the vagus nerve and/or associated neurotransmitter systems. NA, acetylcholine and GABA are the most implicated neurotransmitters related to cognitive enhancement following tVNS. Norepinephrine is the primary neurotransmitter of the ascending arousal system. Acetylcholine is a key neurotransmitter for memory formation with the majority of neurons in the basal forebrain projecting to hippocampal structures and broad cortical regions. GABA is the primary inhibitory neurotransmitter in the brain, although it also plays a key role in shaping and coordinating excitatory neuronal activity. Modulation of the norepinephrine system is hypothesized to be associated with increases in attentional processing, as well as arousal and memory, acetylcholine in memory encoding, and modulation of GABA systems is thought to be associated with sensorimotor function along with working memory and other related cognitive functions; GABA also appears to be a key regulator of resting state networks (Puts et al., 2011; Michels et al., 2012; Unsworth and Robison, 2017).

## Norepinephrine

The primary site of NA synthesis in the brain is the LC. Maintenance of NA and LC integrity has been associated with memory performance and has even been proposed as a cognitive reserve mechanism (e.g., protective against accumulating AD pathology). In cognitively normal older adults, Ciampa et al. (2022) demonstrated a relationship between LC catecholamine synthesis capacity, tau burden and tau accumulation longitudinally. That is, in individuals with high amyloid burden, those with greater catecholamine synthesis capacity in the LC had lower tau burden and accumulation compared to those with lower catecholamine synthesis capacity. LC catecholamine synthesis capacity also moderated memory performance such that higher synthesis capacity was associated with better-than-expected memory performance given an individual’s tau burden. There has been some evidence to suggest increased age itself is associated with a decline in the number of neurons in the LC (particularly the rostral portion), while others have shown that this is only true in brains with evidence of other generalized volume loss and/or neuropathology such as AD (Chan-Palay and Asan, 1989; Manaye et al., 1995). Regardless, given that age is a risk factor for accumulating AD pathology (e.g., amyloid and tau) even in the absence of clinically significant cognitive change, upregulation or maintenance of LC and NA systems may be a route to preservation of aging brain health.

Transcutaneous VNS, specifically auricular stimulation, is thought to engage both tonic and phasic NA arousal. Auricular stimulation (e.g., left external acoustic meatus with left earlobe sham; 8 Hz frequency, 5.0 mA, and 200 pulse width) has been shown in some studies to increase BOLD signal in the LC and network-associated regions (e.g., hippocampus, insula, thalamus) compared to sham stimulation and simultaneously to improve associative memory performance as shown by a face-name learning task (Jacobs et al., 2015, 2020). The mechanism by which tVNS is thought to improve memory performance is by increasing the amount of available NA, thereby upregulating the systems dependent on this neurotransmitter such as memory and attention/arousal. This being said, the demonstrated reliability of auricular tVNS in engaging the LC and NA system is currently low. Some of this may be related to inconsistent or inappropriate outcome measures. Currently used measures of NA system integrity and engagement include pupil dilation, P300 event-related potential (ERP), or salivary alpha-amylase. Other reasons may include poorly validated or inconsistent stimulation protocols. It is also not well known how individual differences in neuroanatomy (e.g., baseline axonal integrity of LC networks) and neurophysiology (e.g., baseline LC integrity) may impact the efficacy of tVNS in affecting LC function.

## Acetylcholine

The nucleus basalis of Meynert (nbM) is the hub of cholinergic neurons in the human brain. Auricular tVNS has been shown to engage the cholinergic system in several ways. Stimulation of the auricular branch of the vagus nerve has downstream effects on the NTS, which, in turn, has widespread projections which include the nbM. It is hypothesized that increased production of acetylcholine via stimulation of the nbM increases memory via its impact on hippocampal functioning (amongst many other cortical areas). However, much of the literature regarding acetylcholine modulation has focused on anti-inflammatory effects in the peripheral and central nervous systems, as discussed in the section on, “Immune and inflammatory mechanisms. Similar to the NA literature, most of the mechanistic studies of tVNS impact on cholinergic function has been conducted in rodent models. Some limitations of this body of literature include widely varying stimulation parameters (e.g., location, timing and electrical waveform characteristics), the type of disease model utilized (e.g., murine model of AD, vascular dementia model, healthy aging), and the outcome measured (e.g., nicotinic receptors, choline acetyltransferase). Noradrenergic pathways are also suggested to have a modulatory influence on cholinergic projections throughout the cortex (Fort et al., 1995). Thus, tVNS may have a direct or indirect (via the LC and NA pathways) effect on cholinergic neurons in the nbM. In neurodegenerative conditions and even healthy cognitive aging, there are alterations in cerebral metabolites including choline-containing compounds (Kakimoto et al., 2016). Choline-containing compounds (Cho) are precursors to acetylcholine and at least one study has shown a moderately positive relationship between Cho levels as measured by magnetic resonance spectroscopy (MRS) and acetylcholine as measured by micro dialysis and high-performance liquid chromatography in a rodent model (Wang et al., 2008). A recent study further showed that these alterations in choline-containing compounds in the parietal region of older adults without cognitive impairment were associated with cognitive performance and cortical volume in regions relevant to maladaptive cognitive aging and neurodegenerative disorders (e.g., hippocampus, parahippocampus, amygdala, anterior cingulate) (Williamson et al., 2021). Other studies have found mixed results relating to levels of Cho in healthy aging individuals depending on the region of the brain assessed (Cleeland et al., 2019). Results suggest dysregulation of acetylcholine production even in healthy cognitive aging.

## GABA

Although there is conflicting evidence regarding GABA, GABA receptors, and GABA-associated networks and their decline with age, GABA’s relationship to cognitive performance has been well-documented in both younger and older adults (Ling et al., 2005; Gao et al., 2013; Marsman et al., 2017). There is some evidence that age-related changes in GABA receptors may result in compensatory changes in GABA-associated networks (Pandya et al., 2019; Ethiraj et al., 2021). Further, several more recent studies using magnetic resonance spectroscopy have demonstrated a negative relationship between levels of frontal GABA and age in cognitively normal older adults (Porges et al., 2017). Transcutaneous VNS may increase the release of GABA by activating the NTS (the NTS projects to the parafacial zone, which is a GABA-ergic region, critical in slow wave sleep). Several fMRI and MRS studies have shown evidence to support this mechanism (Dietrich et al., 2008; Kraus et al., 2013; Frangos et al., 2015; Yakunina et al., 2017). For instance, one study applying continuous 0.25 ms pulses at 25 Hz to the earlobe and left cymba conchae found that the latter produced significant activation of central vagal projections including widespread activity in the ipsilateral NTS (Frangos et al., 2015). However, evidence is limited and sample sizes are frequently small.

## Regional and network activation

There is a widely reported trend of more diffuse brain activation in older individuals. Cabeza et al. (2002) first introduced the Hemispheric Activity Reduction in Older Adults (HAROLD) model to explain this pattern of reduction in hemispheric lateralization particularly in the pre-frontal cortex. Other models of cognitive aging include the right-hemi aging model, which posits that the right hemisphere shows greater age-related decline than the left hemisphere (Dolcos et al., 2002). Additionally, another theory proposed that there is greater recruitment of additional neural circuits in order to meet task demands in the presence of cognitive aging and brain dysfunction (Reuter-Lorenz and Cappell, 2008). Vagus nerve stimulation may serve as a mechanism to engage compensatory neural resources and thus improve cognitive performance.

Kraus and colleagues were among the first to utilize concurrent auricular tVNS and fMRI with 8 Hz frequency and 20 ms pulse width stimulation in the left outer auditory canal in a sample of 22 healthy adults and found increased activation in the insula, pre-central gyrus and thalamus (Kraus et al., 2007). A similar study with stimulation of the inner side of the left tragus in a sample of 4 healthy adults found increased BOLD signal in the left LC and thalamus when compared to a pre-stimulation baseline (Dietrich et al., 2008). Other groups have shown increased functional activation in regions such as bilateral insula, amygdala, thalamus, nucleus accumbens, and frontal cortex while using the left earlobe as a sham stimulation condition (Frangos et al., 2015; Badran et al., 2018). Several of these studies have also reported robust decreases in BOLD signal response during acute tVNS, particularly within the limbic structures and brainstem. Specifically, this has included deactivation in regions such as the hippocampus, parahippocampal gyrus, amygdala, nucleus accumbens, thalamus, and hypothalamus (Kraus et al., 2007; Dietrich et al., 2008; Kraus et al., 2013; Frangos et al., 2015; Frangos and Komisaruk, 2017). Currently, no studies within the literature have investigated resting state network activation in response to tVNS with cognitively healthy older adults. However, several studies have found engagement of networks relevant to healthy cognitive aging such as the Default Mode Network (DMN) (Fang et al., 2016) and Semantic Networks (Murphy et al., 2022) in other populations including older adults with mild cognitive impairment.

The heterogeneity among results reported within available fMRI tVNS studies is likely due in part to methodological differences, particularly pertaining to stimulation parameters (e.g., stimulation frequency, targets, electrode placement). There is substantial variability in device form factors and interface. Non-invasive locations published in the literature include auricular and cervical regions comparisons of these methods are limited. There is a great need for optimization of stimulation parameters to encourage cohesion across future studies and maximize the efficacy of tVNS as a potential intervention. In line with this goal, Yakunina et al. (2017) compared four stimulation locations, the inner tragus, infero-posterior wall of the ear canal, cymba conchae, and earlobe (sham) to determine the most effective location for tVNS. Two 6 min tVNS stimulation runs per electrode location were conducted with monophasic rectangular 500 μs pulses at 25 Hz during resting state fMRI. They found that only the cymba conchae produced a significantly stronger activation in both the NTS and LC compared to sham, with stimulation at the ear canal resulting in the weakest activation of NTS and LC (Yakunina et al., 2017). Another group investigated brainstem fMRI response to auricular tVNS administered at the cymba conchae using four different stimulation frequencies (2, 10, 25, 100 Hz), and found that 100 Hz evoked the strongest response within the left medulla and NTS (Sclocco et al., 2020). Given the role of the vagus nerve and its projections in autonomic functioning, another fMRI study examined how respiratory phase influences the effectiveness of tVNS and found that stimulation induced a more pronounced BOLD signal response during exhalation, with increased activation in the ipsilateral pontomedullary junction (Sclocco et al., 2019). Greater understanding of what accounts for activation differences across tVNS fMRI studies will be imperative for informed interpretation of findings and identifying utility for future intervention development.

It is also important to consider whether brain changes associated with tVNS are consistent with the aging hypotheses proposed by Cabeza and others. Most available studies involving fMRI and tVNS are with younger adults and do not include a cognitive component, limiting the ability to draw conclusions supporting compensation as a mechanism. A few new pilot studies using cervical tVNS have incorporated both fMRI and cognitive tasks but have yielded mixed findings. One group reported simultaneous enhancement of activity within regions associated with attention networks and reduced reaction time during a valenced image anticipation task, while another found improved performance on memory and language tests with concurrent activation within the calcarine gyrus, fusiform gyrus, lingual gyrus, and parahippocampal gyrus (Lerman et al., 2022; Zhang et al., 2022). However, a third study presented opposing findings, as cervical tVNS results in a significant difference in visuospatial performance but did not cause significant changes in neural activity (Klaming et al., 2022). More research incorporating both behavioral and neuroimaging methods within older adults populations is needed to gain a more comprehensive understanding of support for compensation. Regarding maintenance, there are few longitudinal tVNS intervention studies, which limits data to support this mechanism. See Table 1 for summary of fMRI derived network impacts of tVNS.

**Table 1:** fMRI derived network and regional impacts of tVNS

**Study**	**Population**	**Study Design and Intervention**	**Comparator**	**ROIs**	**Outcome**
Kraus et al. (2007)	Study 1: 8 healthy subjects (2 males, 6 females) Study 2: 6 healthy subjects (1 male, 5 females), No range, mean, or SD of ages included	Concurrent tVNS/fMRI; left external acoustic meatus on the inner side of the tragus; pulse width 20 ms, frequency 8 Hz Study 2: three trials of low, high, or alternating low-high stimulation. Study 3: one trial of high earlobe stimulation. In each trial, 4 stimulation periods of 30s followed by a resting period of 1 minute. Low was defined as the threshold of conscious perception for each individual subject. High was defined by the subjective maximum strong sensation that is not painful.	Earlobe (sham; study 3 only)	Whole brain using Talairach coordinates	Increased BOLD signal: insula, precentral gyrus, and thalamus Decreased BOLD signal: amygdala, hippocampus, parahippocampal gyrus, middle and superior temporal gyrus Similar effects across low and high stimulation; low-high alternation did not result in significant activation differences
Dietrich et al. (2008)	4 healthy subjects, aged 26-32 years, mean ± SD: 30 ± 2.7 years	Concurrent tVNS/fMRI; inner side of the left tragus; four alternating stimulation (50s) and baseline (100s) sequences; pulse width 250 μs, frequency 25 Hz	Pre-stimulation baseline	Brainstem, thalamus, prefrontal cortex, post-central gyrus, nucleus caudatus, amygdala, hippocampus, putamen, anterior cingulum	Increased BOLD signal: left locus coeruleus, thalamus (left>right), left prefrontal cortex, right and left postcentral gyrus, left posterior cingulated gyrus and left insula Decreased BOLD signal: right nucleus accumbens, right cerebellar hemisphere
Kraus et al. (2013)	16 healthy subjects, aged 20-37 years, no mean or SD included	Concurrent tVNS/fMRI; 8 subjects received stimulation at the anterior wall while 8 received stimulation at the posterior side of the left outer auditory canal; pulse width 20 ms, frequency 8 Hz	Earlobe (sham)	Locus coeruleus, thalamus, prefrontal cortex, postcentral gyrus, posterior cingulate gyrus, insula, nucleus accumbens, cerebellum, nuclei of the vagus nerve	Decreased BOLD signal: parahippocampal gyrus, posterior cingulate cortex, right thalamus (pulvinar), locus coeruleus, the solitary tract
Frangos et al. (2015)	12 healthy subjects, aged 21-71 years, mean ± SD: 32.6 ± 13.8 years	Concurrent tVNS/fMRI; 7-min left cymba conchae stimulation; continuous 0.25 ms pulses, frequency 25 Hz	Earlobe (sham)	Lower brainstem, amygdala, hippocampus, insula, nucleus accumbens, thalamus, hypothalamus, paracentral lobule of the cerebral cortex	Increased BOLD signal: ipsilateral nucleus of the solitary tract, bilateral spinal trigeminal nucleus, dorsal raphe, locus coeruleus, and contralateral parabrachial area, amygdala, nucleus accumbens, bilateral activation of the paracentral lobule Decreased BOLD signal: bilateral hippocampus and hypothalamus
Frangos and Komisaruk (2017)	13 healthy subjects, aged 20-29 years, mean ± SD: 23.1± 3.3 years	Concurrent tVNS/fMRI; transcutaneous electrical stimulation applied for 2 min to the right antero-lateral surface of the neck; 5 sinusoidal wave pulses at 5000 Hz repeated at frequency of 25 Hz	Right postero-lateral surface of the neck (control)	Whole brain, brainstem	Increased BOLD signal: nucleus of the solitary tract, parabrachial area, primary sensory cortex, frontal cortex, basal ganglia, insula Decreased BOLD signal: hippocampus, visual cortex, and spinal trigeminal nucleus
Yakunina et al. (2017)	37 healthy subjects, mean ± SD: 30.9± 8.2 years	Concurrent tVNS/fMRI; left inner tragus, inferoposterior wall of the ear canal, cymba conchae; two 6-min tVNS stimulation runs per electrode location; monophasic rectangular 500 μs pulses, frequency 25 Hz	Earlobe (sham)	Locus coeruleus, nucleus of the solitary tract	tVNS at cymba conchae results in strongest activation of locus coeruleus and nucleus of the solitary tract
Badran et al. (2018)	17 healthy subjects, aged 18-45 years, mean ± SD: 25.8 ± 7.59 years	Concurrent tVNS/fMRI; auricular tVNS applied to left tragus for 60 seconds on, 60 seconds off; pulse width 500 μs, frequency 25 Hz	Earlobe (sham)	(Cluster locations) Central operculum, postcentral gyrus, insula, angular gyrus, inferior frontal gyrus, supplementary motor area, superior frontal gyrus, cerebellum, caudate	Increased BOLD signal: right caudate, bilateral anterior cingulate, cerebellum, left prefrontal cortex, mid-cingulate gyrus
Sclocco et al. (2019)	16 healthy adult subjects, mean ± SD: 27 ± 6.6 years	Concurrent tVNS/fMRI; auricular left tVNS applied to cymba conchae during exhalation (eRAVANS) or inhalation (iRAVANS); frequency 25 Hz, pulse width 450 μs	Exhalation-gated stimulation over greater auricular nerve/earlobe (control)	Dorsal medulla, bilateral locus coeruleus, and dorsal and median raphe nuclei	eRAVANS evoked fMRI signal increase in ipsilateral pontomedullary junction in a cluster including purported NTS
Sclocco et al. (2020)	30 healthy subjects, mean ± SD: 29 ± 9.8 years	Concurrent tVNS/fMRI; auricular RAVANS applied to cymba conchae; four stimulation frequencies (2, 10, 25, 100 Hz), pulse width 300 μs	Cymba conchae with no current (sham)	Bilateral locus coeruleus, dorsal and median raphe nuclei, and periaqueductal gray	RAVANS delivered at 100 Hz evoked the strongest brainstem response
Lerman et al. (2022)	24 healthy subjects, aged 18-54 years, mean ± SD: 27 ± 8.4 years	tVNS applied for 2 minutes to the right anterior cervical area on the neck; 5 sinusoidal wave pulses at 5000 Hz repeated at frequency of 25 Hz fMRI collected 40 minutes later during completion of continuous performance task and cued valence anticipation task	Right lateral cervical area on the neck (sham)	Whole brain; BOLD response examined to identify neural regions that demonstrate group (tVNS, sham) and task effects	tVNS subjects demonstrated increased activation to negative valence image anticipation and decreased to positive valence image anticipation in the left precentral gyrus, left superior frontal gyrus, left anterior insula, left anterior cingulate, left post-central gyrus
Zhang et al. (2022)	21 healthy students, aged 20-22 years, mean ± SD: 21.38 ± 0.962 years	Concurrent tVNS/resting state fMRI applied for 8 minutes to the left cervical vagal nerve; pulse width 0.5 ms, frequency 25 Hz Randomized crossover-controlled trial; one group received tVNS then sham; another group received sham then tVNS	Left cervical vagal nerve with lower voltage (sham)	Somatosensory areas, nucleus tractus solitarius, limbic lobe, amygdala, hippocampus, insula, precentral gyrus, thalamus, hypothalamus, anterior cingulate cortex, left dorsolateral and prefrontal cortex	Increased BOLD signal: calcarine gyrus, fusiform gyrus, lingual gyrus, and parahippocampal regions
Klaming et al. (2022)	30 healthy subjects, aged 18-54 years, mean ± SD: 27 ± 8.4 years	tVNS applied for 2 minutes to the right anterior cervical area of the neck; 5000 Hz repeated at frequency of 25 Hz fMRI collected 35 minutes later during completion of a matrix reasoning task	Right lateral cervical area on the neck (sham)	Unspecified; BOLD response examined to identify neural regions that demonstrate group (tVNS, sham) and task effects	No difference between tVNS and sham groups on BOLD response

## Immune and inflammatory mechanisms

The relationship between vagus nerve activity and inflammation was proposed by Tracey in the early 2000s (Tracey, 2002; Tracey, 2009). This relationship was primarily hypothesized to take place via the cholinergic anti-inflammatory pathway (CAIP) and is thought to involve both efferent and afferent projections of the vagus nerve. Specifically, efferent communication from the vagus nerve to the dorsal motor nucleus to the celiac ganglion may reduce peripheral cytokines through the CAIP. The vagus nerve plays a key role in regulating peripheral cholinergic release, which impacts release of inflammatory markers associated with increased immune response, such as tumor-necrosis factor (TNF), interleukin-1 (IL-1), high mobility group box 1 (HMGB1), and other cytokines via the CAIP. Additionally, tVNS afferent projections to the HPA axis may play a role in the anti-inflammatory response (i.e., reducing stress response, improving autonomic function) and thus improve cognition. Chronic inflammatory states and mental illnesses with HPA axis involvement, such as depression and post-traumatic stress disorder (PTSD), are associated with cognitive decline and increased risk for dementia (Byers and Yaffe, 2011; Richard et al., 2013; Günak et al., 2021). Thus, stimulation of the vagus nerve and subsequent downstream modulation of inflammatory pathways via the efferent and afferent pathways may be a means of improving brain health. As described below, a handful of studies, the majority of which were in animal models, have explored the impact of tVNS/VNS on peripheral and central immune functioning and have found promising results.

Huffman et al. (2019) found anti-inflammatory effects of minimally-invasive percutaneous VNS (pVNS) in a mouse model of cognitive dysfunction due to lipopolysaccharide (LPS) endotoxemia. Both LPS conditions were randomly assigned to receive 10 or 20 Hz pVNS and both showed reduced hippocampal TNF-*α* levels relative to the LPS only group, demonstrating modulation of a proinflammatory cytokine (TNF-*α*) with minimally-invasive pVNS. Subramanian et al. (2020) measured gene expression in brainstem nuclei known to have connections with the vagus nerve, including the spinal trigeminal nucleus (SP5) and subfornical organ (SFO), before and after tVNS in an animal model with inflammation introduced via a high salt diet. Stimulation was applied under isoflurane anesthesia to the concha region via a TENS device for 30 min daily over 4 weeks with 20 Hz frequency, 0.2 ms pulse duration, and 2 mA amplitude. Stimulation induced reduced gene expression of IL-6, a pro-inflammatory cytokine, in the SP5 and reduced IL-1β, another pro-inflammatory cytokine, in the SFO following tVNS.

Another study found evidence for divergent modulatory effects of afferent and efferent vagus projections on inflammation. In this animal model, wild type and ChAT (choline acetyltransferase) knockout mice were treated with invasive stimulation to either afferent or efferent pathways of the vagus (Murray et al., 2021). Though efferent VNS suppressed cytokine production in response to LPS in wildtype mice, the LPS-treated ChAT knockout mice did not show the same effect. With afferent stimulation, both the wildtype and ChAT mice showed a suppression of cytokines in response to LPS. Additional mice were treated with a Beta-2-adrenergic receptor antagonist. After comparing efferent and afferent stimulation in these mice, neither showed a significant reduction in pro-inflammatory cytokines. These findings suggest that efferent but not afferent VNS is dependent upon T-cell derived acetylcholine (Ach) and that both efferent and afferent vagus mechanisms utilize signaling through Beta-2-adrenergic receptors. Cai et al. (2019) tested the effects of tVNS on α7nAchR expression, TNF-*α*, IL-1*β*, NF-*κ*B, and apoptosis in the hippocampus of a rat model of post-operative cognitive dysfunction (POCD) following a laparotomy. While the laparotomy did not affect expression of anti-inflammatory hippocampal *α*7nAch receptors, tVNS increased their expression. In addition, rats in the tVNS-treated POCD group had lower levels of the proinflammatory cytokines TNF-*α* and IL-1*β* than the POCD only group. Surgical alterations of NF-κB, a proinflammatory transcription factor, were also reduced in the tVNS and POCD group versus the laparotomy only group. Finally, the tVNS and POCD group had an elevated ratio of anti-apoptotic proteins (Bcl-2) to pro-apoptotic proteins (Bax) compared to the POCD only group. Lerman et al. (2016) found decreased blood levels of peripheral inflammatory markers after 24 h in healthy younger and middle-aged adults (humans) (*N* = 20) who received tVNS compared to those who received sham. Specifically, the tVNS group showed a pattern of decreased cytokines (e.g., TNF, IL-1*β*) and chemokines (e.g., MCP-1, IL-8) at 24-h post-stimulation. Participants received either sham or active stimulation with the gammaCore stimulation device. Stimulation in the active group was applied cervically at 25 Hz. Amplitude was adjusted by the user with a maximum current of 24 V and 60 mA and stimulation was applied for 2 min (i.e., 30 s ramp period and 90 s of actual stimulation).

As a part of the neuroendocrine immune axis, the vagus nerve may also impact cognition through modulation of microglia. Chronic activation of microglia has been shown to be associated with worse cognition and is associated with progression of AD pathology (Malpetti et al., 2020). Previous research also suggests that chronic activation of microglia may be reversible through stimulation of the vagus nerve by returning microglia to a healthy state via locus coeruleus norepinephrine upregulation, which activates beta-receptors on microglia. In a murine mouse model of AD, non-invasive vagus nerve stimulation of the neck was shown to affect microglia such that older APP/PS1 genotype mice, but not younger mice, had beneficial morphological changes (e.g., longer branch length, greater number of branches) (Kaczmarczyk et al., 2018). In addition to the effects of pVNS on TNF-*α* discussed previously, LPS injection in these mice also changed the morphology of microglia in the hippocampus, such that microglial cells became non-ramified, retracted their ramifications, and increased their soma volume (Huffman et al., 2019). LPS mice treated with pVNS at 10 or 20 Hz had less non-ramified microglia compared to the LPS only group. Only 10 Hz pVNS had more ramified microglia compared to the LPS only group. LPS-treated mice receiving both 10 and 20 Hz pVNS also had reduced CD68 immunoreactivity, a protein expressed in high levels in activated microglia, compared to LPS only mice (Chistiakov et al., 2017). These results suggest that pVNS affected immune functioning in the context of LPS via modulation of TNF-*α* and microglial morphology and activation. While most studies did not investigate cognition in the context of vagal modulation of immune functioning, Huffman and colleagues also found that pVNS restored memory dysfunction in LPS-treated mice, particularly for “where” and “when” aspects of episodic memory, to levels of control mice (Huffman et al., 2019). These results demonstrate both the ability of pVNS treatment to rescue memory deficits due to LPS as well as its potential mechanism of action via modulation of immune function.

## tVNS and cognitive outcomes in adults

Several studies have investigated the impact of tVNS on cognition in older adults with cognitive impairment (e.g., MCI and dementia) and have found evidence for improved test performance after stimulation. For example, a recent clinical trial by Wang et al. (2022) found improvements on a broad cognitive screener (MoCA-Basic) and a measure of verbal memory (AVLT-HuaShan version) in the stimulation group (left auricular cavum conchae, 0.3 ms square wave pulses, 20 Hz, for 30 s, repeated every 5 min) compared to sham. Though these are meaningful results, it may be even more important to determine the efficacy of early intervention in healthy adults to prevent or delay cognitive decline.

Improvements in cognitive performance have been demonstrated in younger and older healthy adults, even after a single session of tVNS. At least one study has found that, after only a single session of tVNS, performance on a face-name association task (*η*^2^ = 0.144) and an episodic memory task improved (*η*^2^ = 0.167) in a sample (mean age = 61) of 30 healthy older adults (Jacobs et al., 2015). Another study of 32 younger healthy adults (mean age = 23) found that tVNS, administered to the cymba conchae of the left ear with a pulse width of 200–300 μs at 25 Hz and an on–off cycle of 30 s, was associated with improvements in cognitive flexibility (*d* = 0.494) on a set-shifting paradigm (Borges et al., 2020). Other studies have found improvements in emotion recognition (Colzato et al., 2017), divergent thinking (Colzato et al., 2018), and attentional processing (Ventura-Bort et al., 2018). However, not all studies have found large improvements. A recent meta-analysis including 19 total studies found a small overall weighted effect size (hedge’s g = 0.21, CI = 0.21–0.29, *t* = 4.99, *p* < 0.0001) with mixed results for domain-specific effect-sizes (memory = 0.12, *p* = 0.51; attention = 0.39, *p* = 0.13; executive function = 0.66, 95% CI = 0.14–1.17, *t* = 2.88, *p* = 0.02) (Ridgewell et al., 2021).

## Additional considerations

Well-characterized sleep changes in older age include difficulty with initiating and maintaining sleep and decreased proportion of slow-wave and rapid-eye movement sleep. Poor sleep quality and other sleep interruptions are related to incidence of neurodegeneration and poor cognitive outcomes (Bliwise, 2004; Lim et al., 2013). Specifically, tau progression is associated strongly with the degree of sleep disruption. Thus, interventions targeting sleep may improve cognitive functioning and/or slow degenerative processes in the CNS. Several therapeutics have been investigated with promising results, including bright light therapy (van Maanen et al., 2016), hypnotics (la et al., 2019), melatonin (Cruz-Aguilar et al., 2018), and continuous positive airway pressure devices (Jorge et al., 2019). tVNS may also prove helpful for improving sleep via pathways through the NTS, which is critical in regulating sleep. As sleep onset occurs, parasympathetic vagal tone increases as sympathetic tone decreases (Jafari, 2017). Further, as sleep progresses from stage 1 to deeper non-REM sleep, parasympathetic tone continues to increase. If sleep is disordered, as it is in many neurological and neuropsychiatric disorders, such as MCI, AD, and PTSD, these stages are often affected (Peter-Derex et al., 2015; D'Rozario et al., 2020). In certain neuropsychiatric disorders, such as PTSD, this is thought to be due to a dysregulation of the noradrenergic system. PTSD is associated with onset of MCI/AD and APOE4, a genetic risk factor for AD, and has been shown to moderate the relationship between combat exposure and PTSD symptoms (D'Rozario et al., 2020). As VNS and tVNS have been shown to possibly increase parasympathetic tone and decrease sympathetic tone, interventions using tVNS may improve sleep and thus affect cognitive aging trajectory.

Mood disturbances also are well known to exacerbate cognitive dysfunction and facilitate cognitive decline (Brendel et al., 2015; MacQueen and Memedovich, 2017). For example, PTSD is associated with a 55% greater risk for all-cause-dementia, while several meta-analyzes have demonstrated that depression confers an approximately 2-fold greater risk of development of dementia (Barnes et al., 2012; Brendel et al., 2015; Cherbuin et al., 2015; Kuring et al., 2018; Günak et al., 2021). VNS devices are FDA-approved for treatment of depression, and research on tVNS suggests it may also be effective at reducing self-reported depressive symptoms as well as symptoms of PTSD such as hyperarousal (Carreno and Frazer, 2017; Lamb et al., 2017). As a result, tVNS may be able to improve cognitive aging via providing symptom relief to decrease risk of future development of cognitive decline.

## Future directions

Transcutaneous vagus nerve stimulation is a safe, well-tolerated, and versatile potential intervention for a range of age-associated disorders and may also be useful for age-related cognitive changes. The current body of research available for mechanisms in healthy aging suggests it may be useful as a primary intervention; however, its ease of use also allows for tVNS application in conjunction with other interventions. For example, tVNS can easily be applied during cognitive training, cognitive rehabilitation, exercise, behavioral therapy, or with other neuromodulation interventions. It can also be applied to treat symptoms of other disorders (e.g., depression or insomnia) that impact cognition and increase risk of cognitive decline. As the field continues to grow, standardization of practice becomes increasingly important to allow for data comparisons across studies. A recent consensus paper from Farmer et al. (2021) highlights important considerations for future tVNS research, including minimal reporting standards for manuscripts and recommendations for participant inclusion/exclusion, potential confounding variables (e.g., age, sex, medical conditions, ear anatomy, time of day, control condition), outcome parameters, potential biomarkers, and commonly used stimulation parameters. Each of these considerations remain important when integrating tVNS with other interventions.

## Author contributions

JW: Initial conceptualization, writing, and editing. ET: conceptualization, writing, and editing. EP and DL: concept and editing AO’N, JC, and SO: literature review and writing. DS: literature review, editing, and writing. All authors contributed to the article and approved the submitted version.

## Funding

This work was supported by the NIH (NIH R21AG054876) and Veteran’s Affairs (VAMC I0RX003140) McKnight Brain Research Foundation.

## Conflict of interest

JW, EP, and DL are on the scientific advisory board for Evren Technologies, a company developing a closed loop transcutaneous vagus nerve stimulation device based on a patent held by the University of Florida (JW, DL, and EP inventors). EP, DL, and JW have an additional patent under development via the University of Florida related to an implementation technology associated with vagus nerve stimulation. No resources from Evren were used in the current study. No studies involving Evren technology were reviewed.

The remaining authors declare that the research was conducted in the absence of any commercial or financial relationships that could be construed as a potential conflict of interest.

## Publisher’s note

All claims expressed in this article are solely those of the authors and do not necessarily represent those of their affiliated organizations, or those of the publisher, the editors and the reviewers. Any product that may be evaluated in this article, or claim that may be made by its manufacturer, is not guaranteed or endorsed by the publisher.
